# Supplementary material for: Non-linear interaction between physical activity and polygenic risk score of body mass index in Danish and Russian populations
Source: PLoS One. 2021 Oct 18;16(10):e0258748. doi: 10.1371/journal.pone.0258748 (PMC8523041; doi:10.1371/journal.pone.0258748)
Supplement: S1 File — (ZIP) [file pone.0258748.s001.zip › Supplementary File 1/!ReadMe.docx]

**S1 File**

**Distributions of the BMI-associated phenotypes of individuals, stratified by BMI polygenic risk score (PRS) deciles.**

List of phenotypes:

- Dyslipidemia markers: total cholesterol, high-density lipocholesterol, triglycerides,
- Inflammation markers: C-reactive protein (high sensitive hs-CRP), interleukin-18 (IL18),
- Cardiovascular diseases markers: diastolic blood pressure, systolic blood pressure, pulse pressure,
- Blood sugar level markers: hemoglobin A1C, fasting glucose, 30 min & 120 min glucose during oral glucose tolerance test (OGTT),
- Insulin sensitivity or resistance markers: homeostatic model assessment of insulin resistance (HOMA-IR), fasting insulin,
- Satiety markers: leptin (LEP),
- Anthropometrics: hip, waist circumference, waist-to-hip ratio, weight, height.

All graphs are cut at 10% / 90% quantiles on the y-axis. Box plots represent median and 25% / 75% quantiles. Green lines show trend and 95% confidence interval for the regression between the trait and the decile as numeric variable. Data shown are unadjusted for age and sex.
