# Supplementary figures and images for: Non-linear interaction between physical activity and polygenic risk score of body mass index in Danish and Russian populations
Source: PLoS One. 2021 Oct 18;16(10):e0258748. doi: 10.1371/journal.pone.0258748 (PMC8523041; doi:10.1371/journal.pone.0258748)

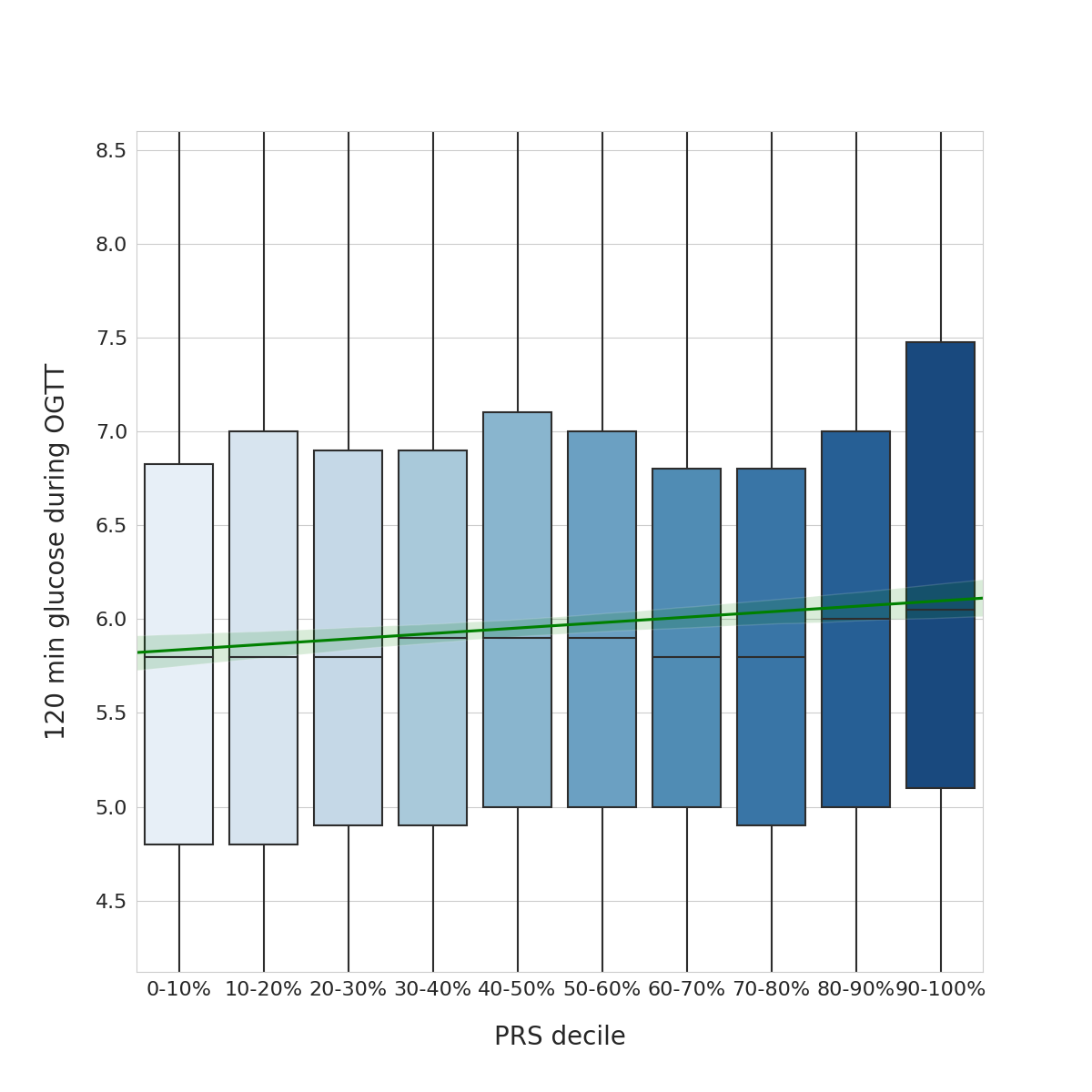

Supplement: S1 File — (ZIP) [file pone.0258748.s001.zip › Supplementary File 1/120 min plasma glucose during OGTT.png]

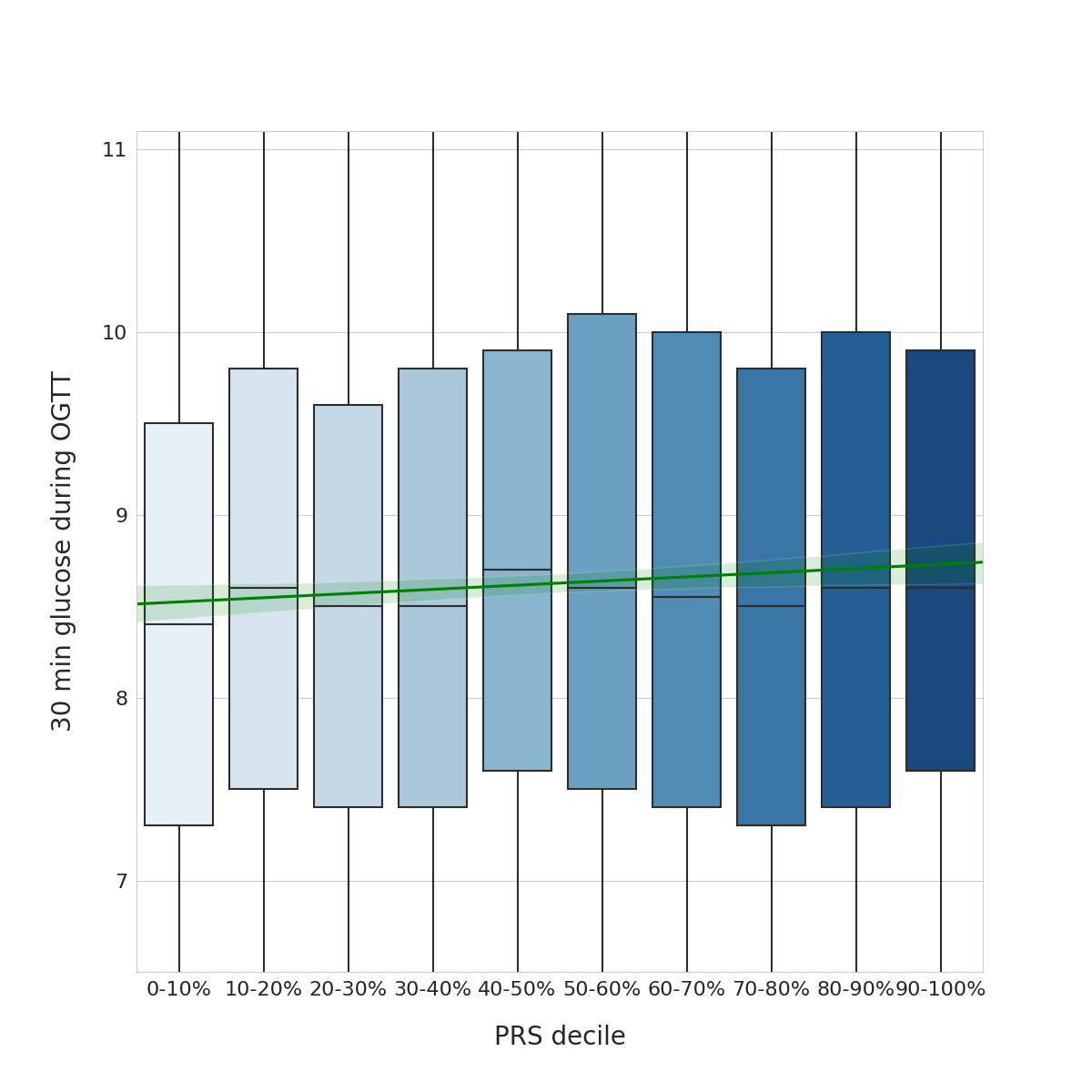

Supplement: S1 File — (ZIP) [file pone.0258748.s001.zip › Supplementary File 1/30 min plasma glucose during OGTT.png]

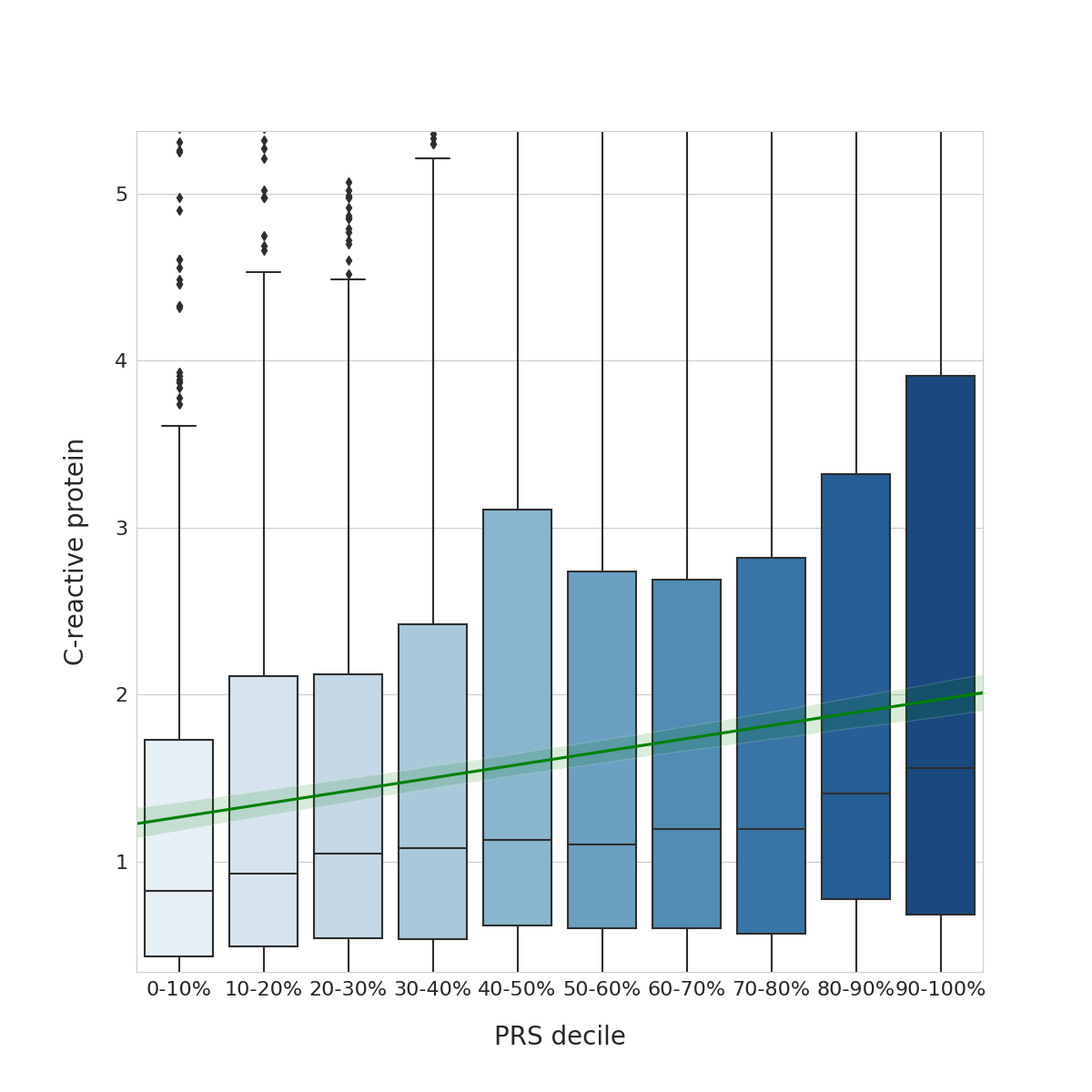

Supplement: S1 File — (ZIP) [file pone.0258748.s001.zip › Supplementary File 1/Serum C-reactive protein.png]

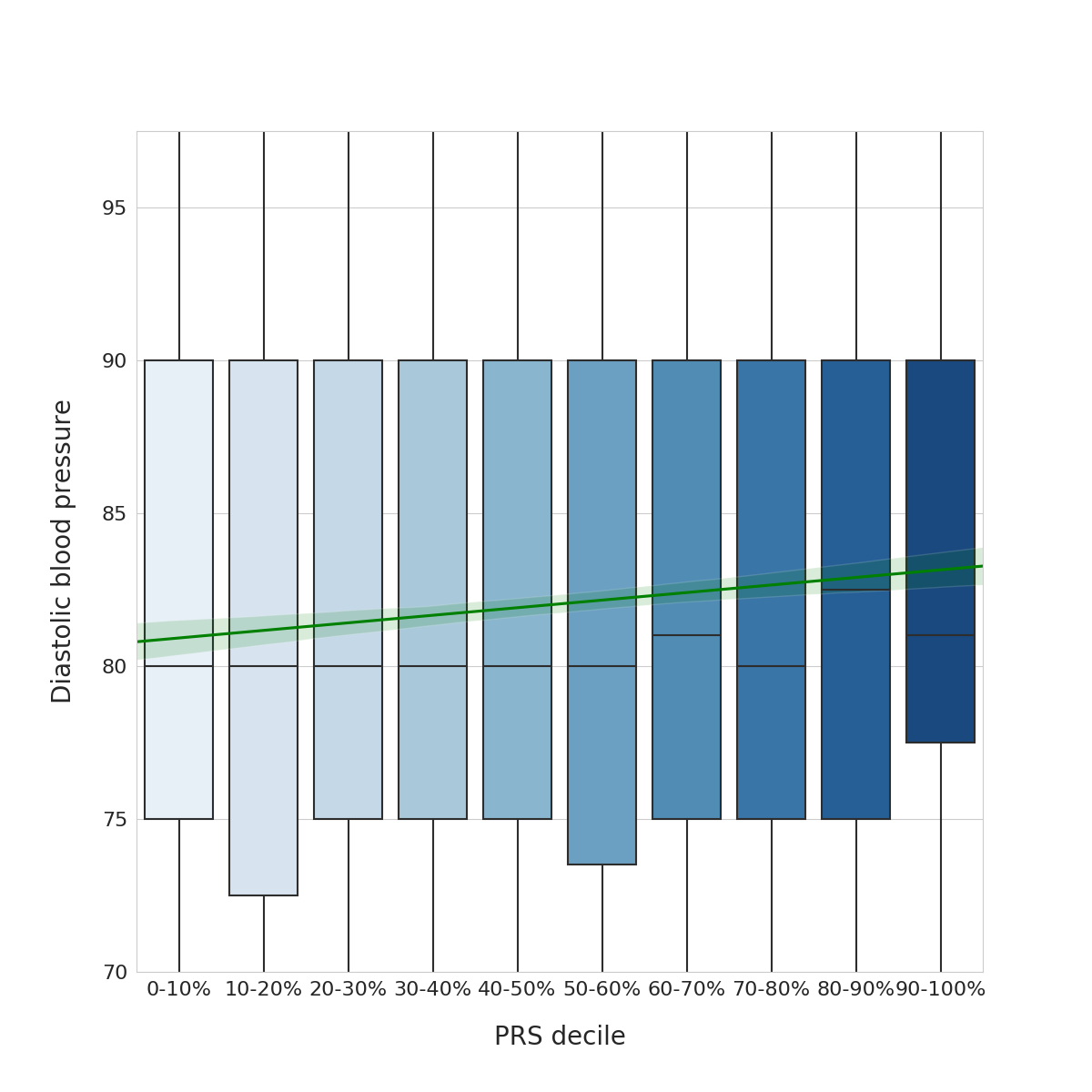

Supplement: S1 File — (ZIP) [file pone.0258748.s001.zip › Supplementary File 1/Diastolic blood pressure.png]

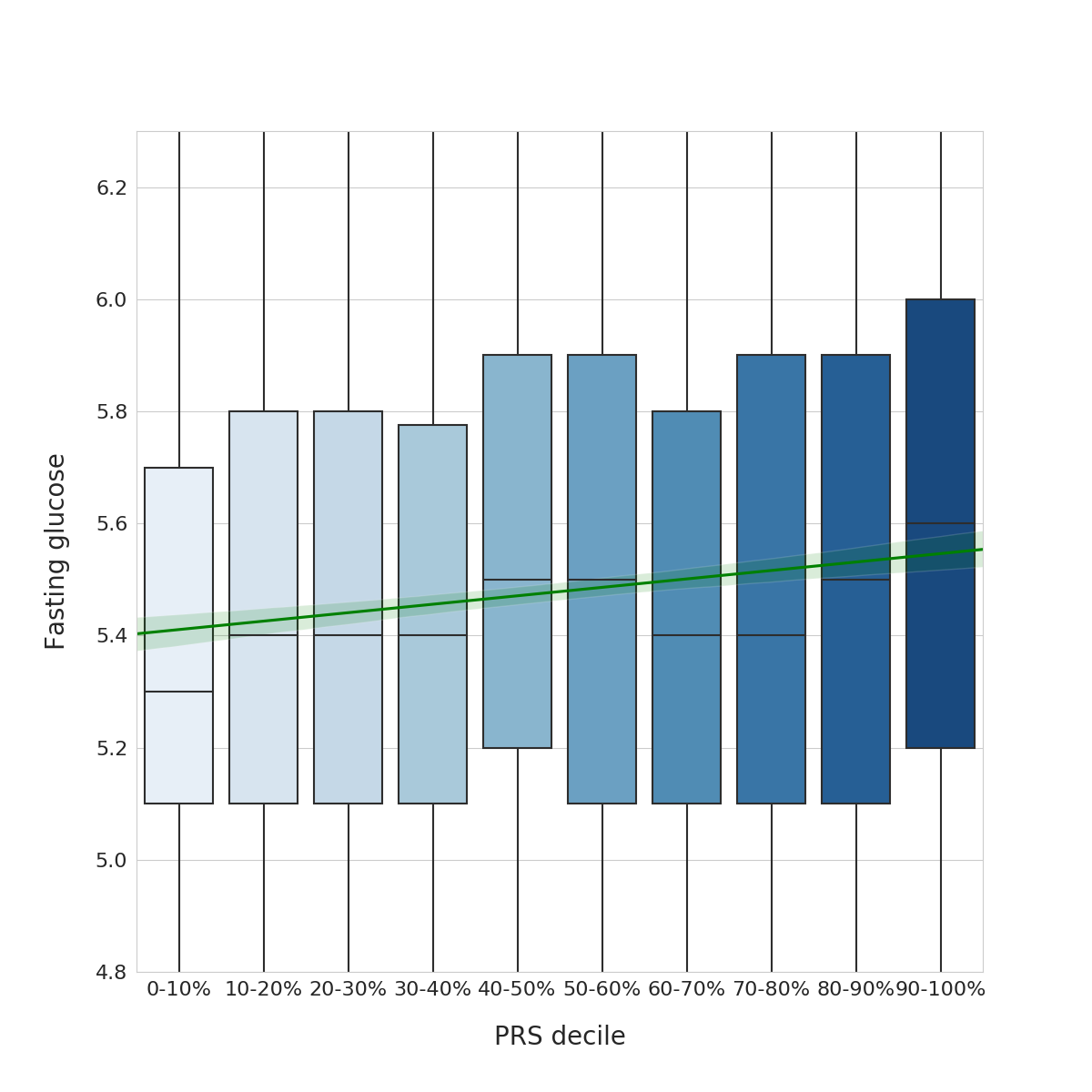

Supplement: S1 File — (ZIP) [file pone.0258748.s001.zip › Supplementary File 1/Fasting plasma glucose.png]

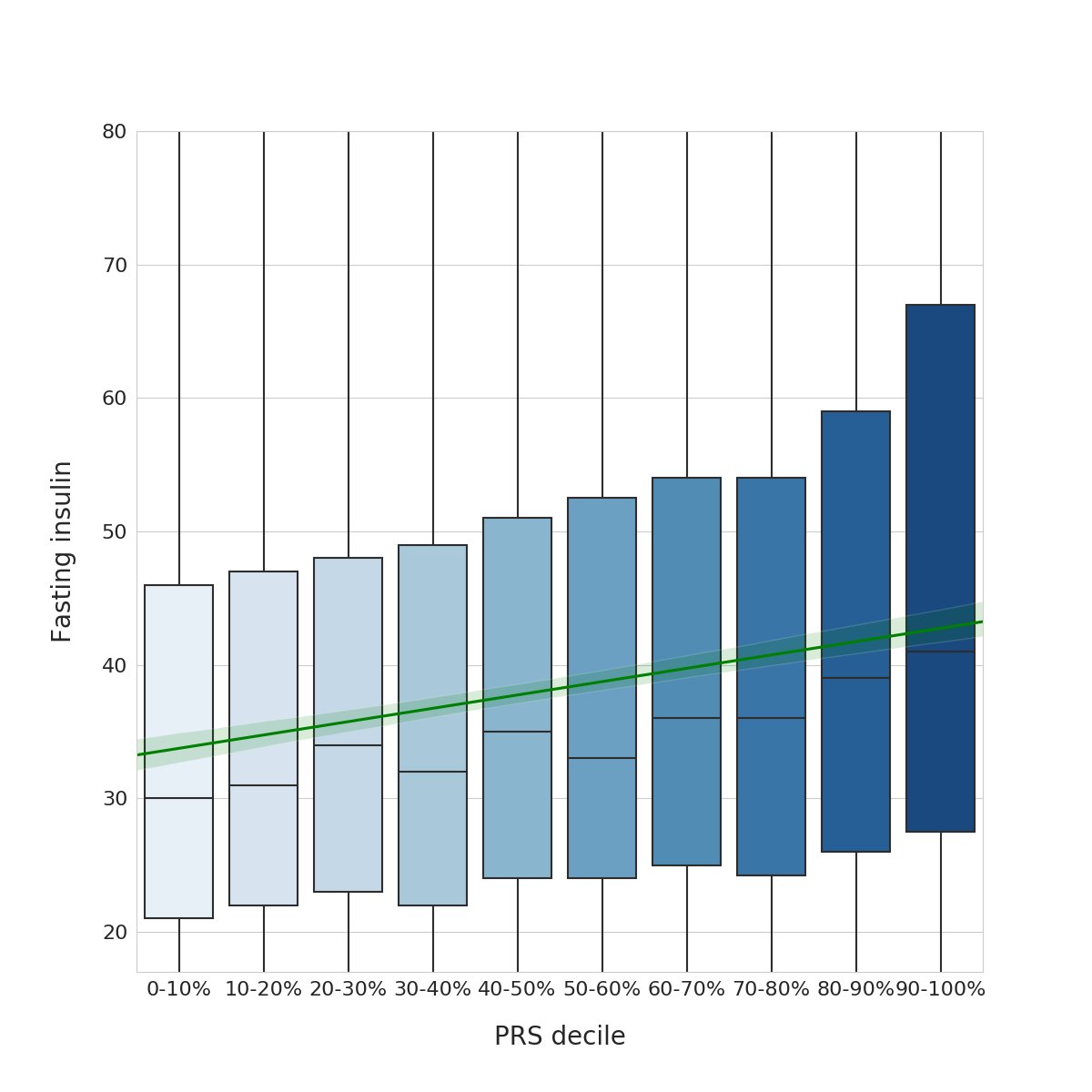

Supplement: S1 File — (ZIP) [file pone.0258748.s001.zip › Supplementary File 1/Fasting serum insulin.png]

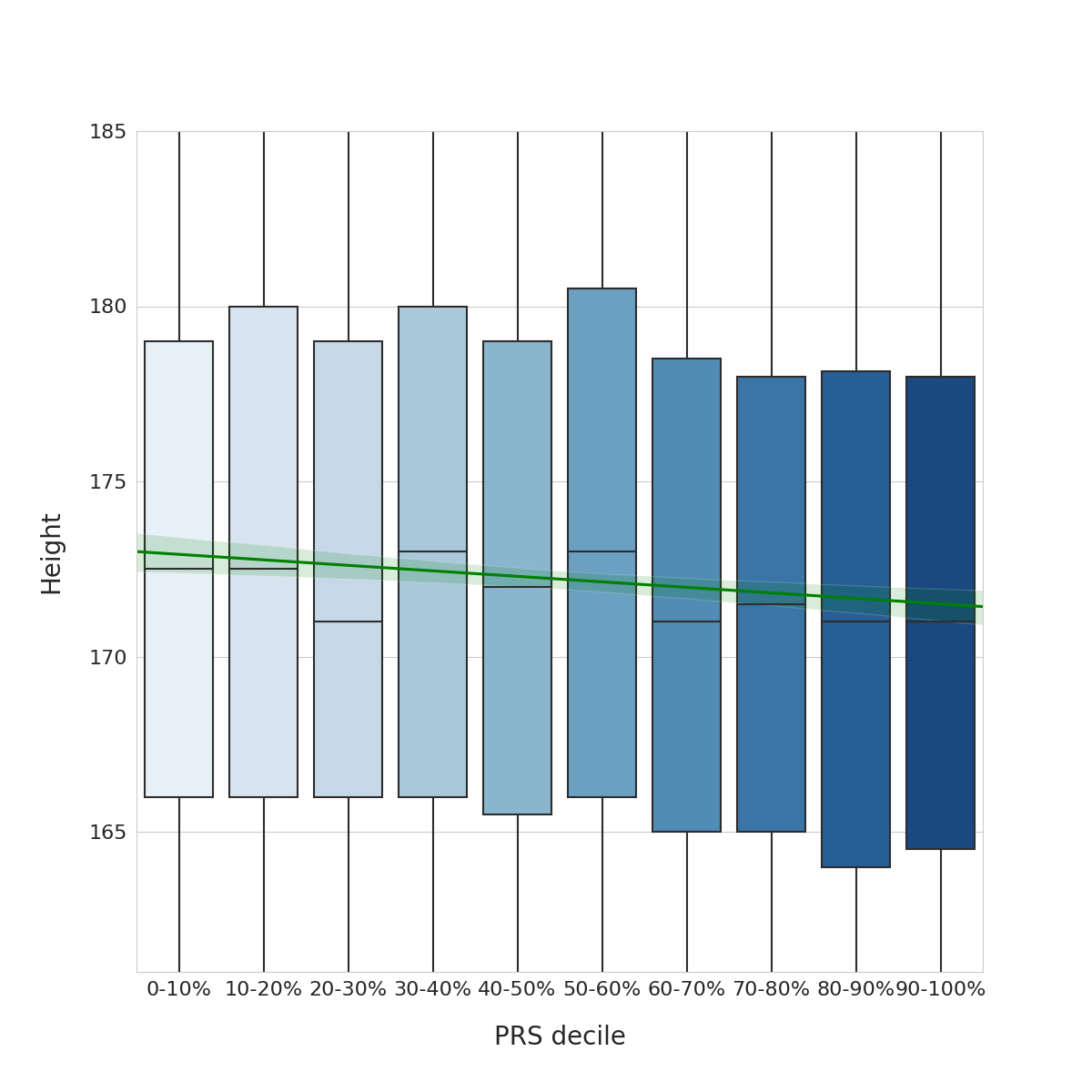

Supplement: S1 File — (ZIP) [file pone.0258748.s001.zip › Supplementary File 1/Height.png]

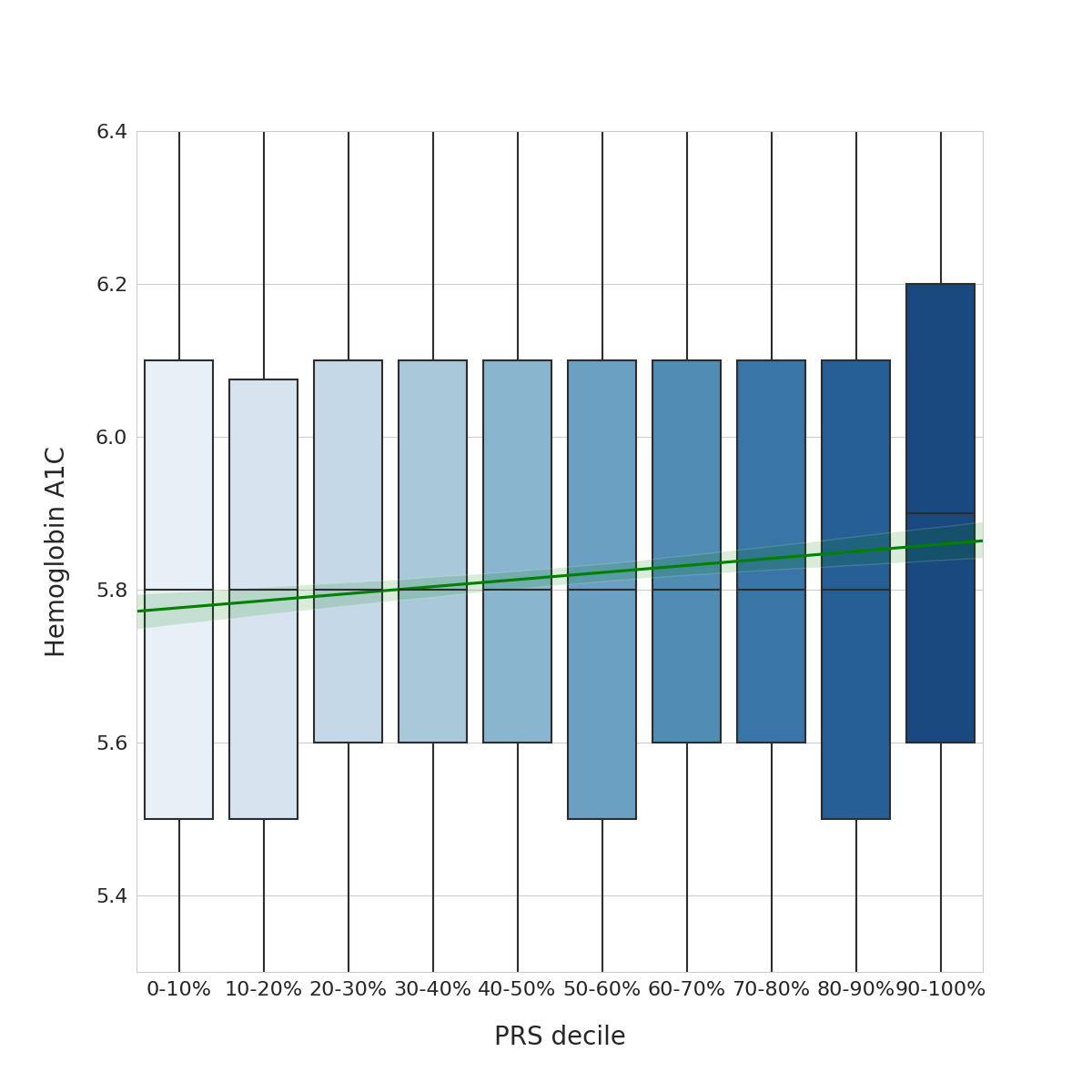

Supplement: S1 File — (ZIP) [file pone.0258748.s001.zip › Supplementary File 1/Hemoglobin A1C.png]

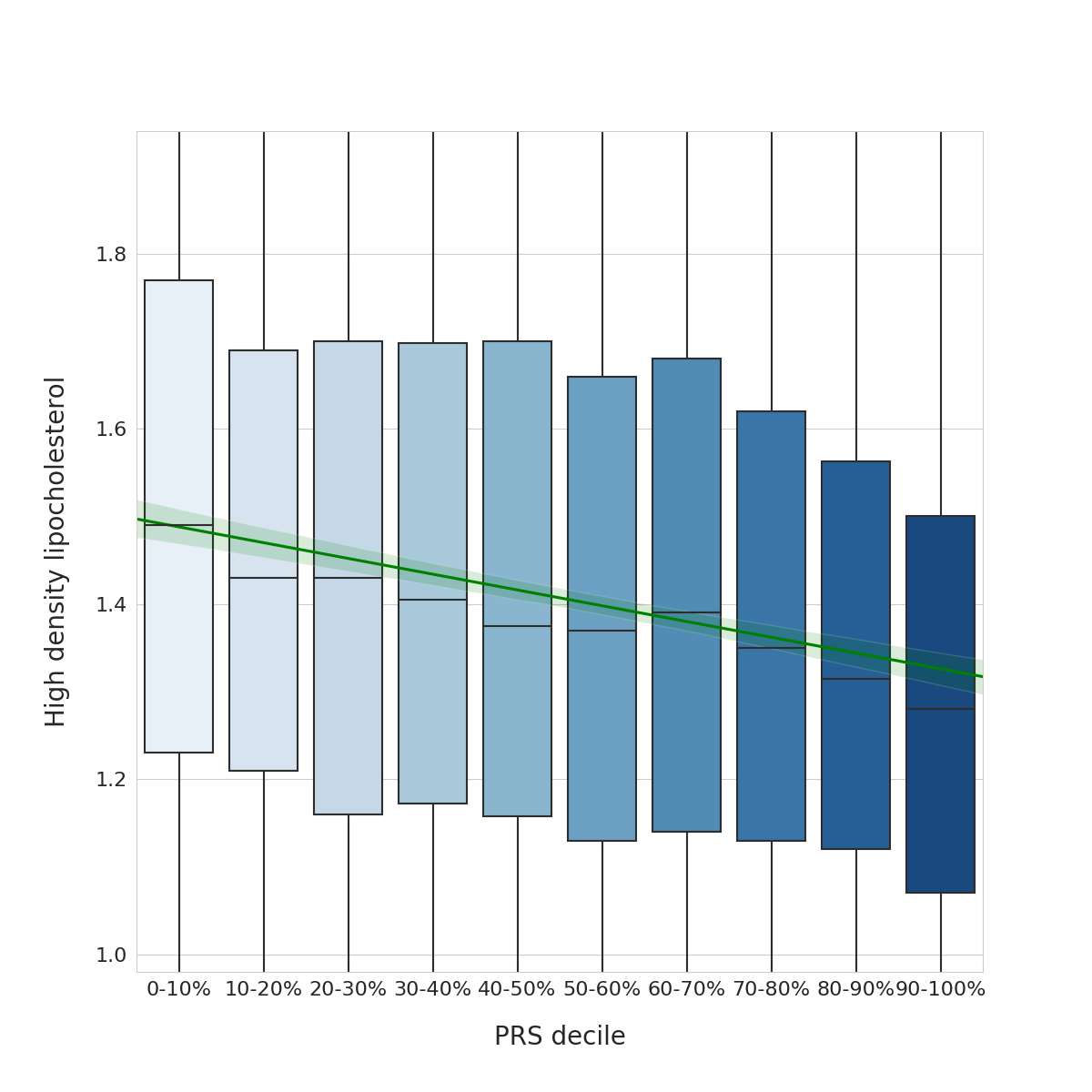

Supplement: S1 File — (ZIP) [file pone.0258748.s001.zip › Supplementary File 1/High density lipocholesterol.png]

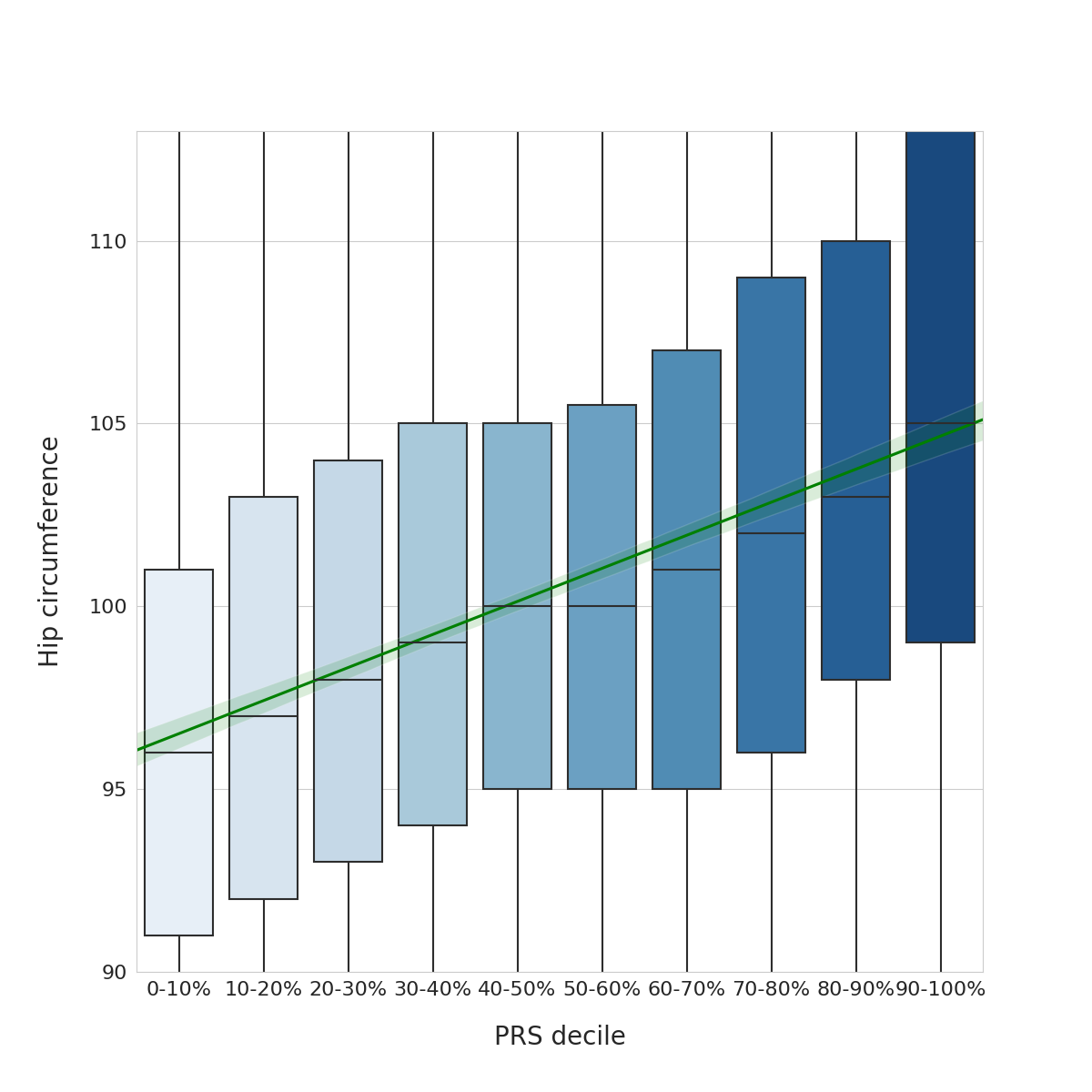

Supplement: S1 File — (ZIP) [file pone.0258748.s001.zip › Supplementary File 1/Hip circumference.png]

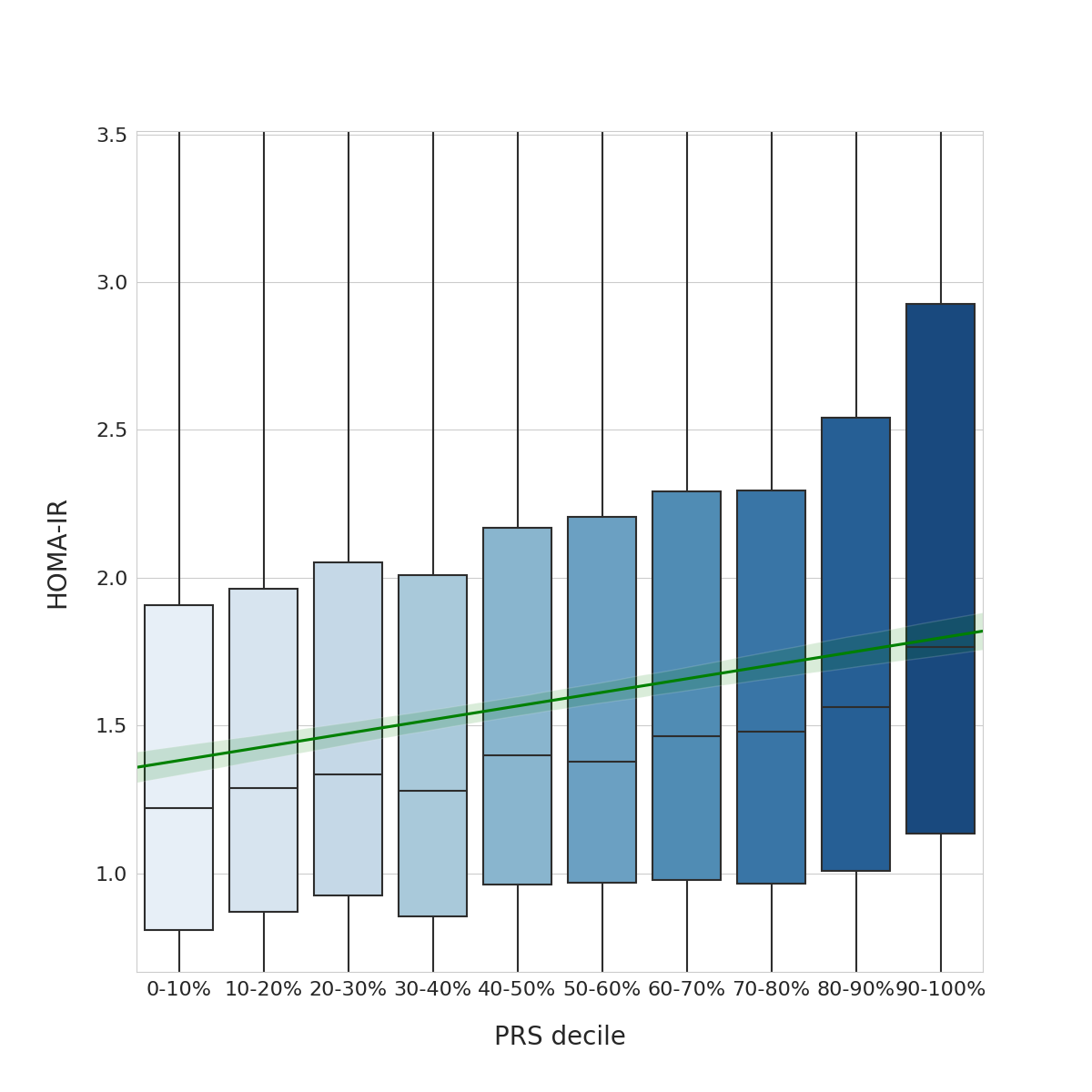

Supplement: S1 File — (ZIP) [file pone.0258748.s001.zip › Supplementary File 1/HOMA-IR.png]

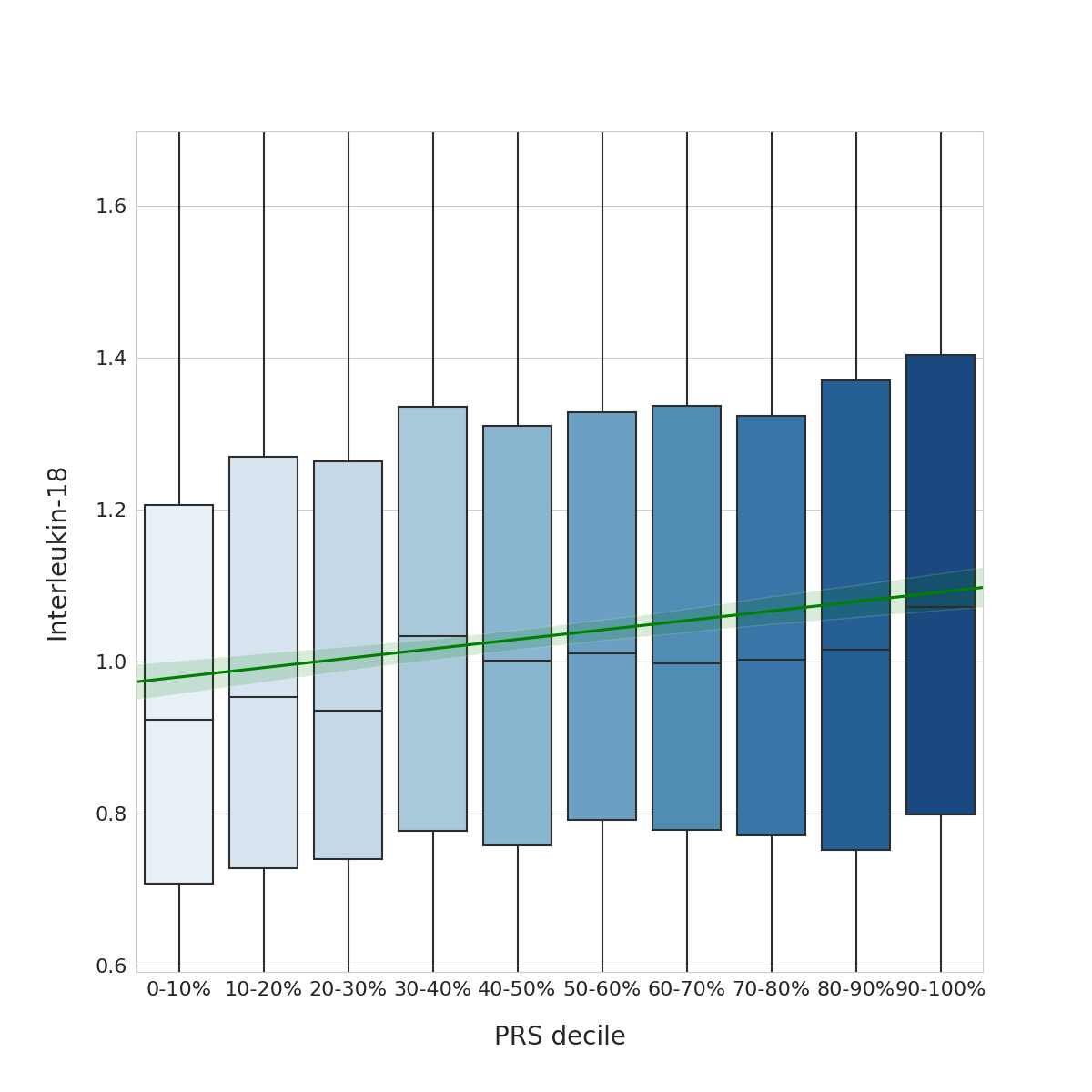

Supplement: S1 File — (ZIP) [file pone.0258748.s001.zip › Supplementary File 1/Interleukin-18.png]

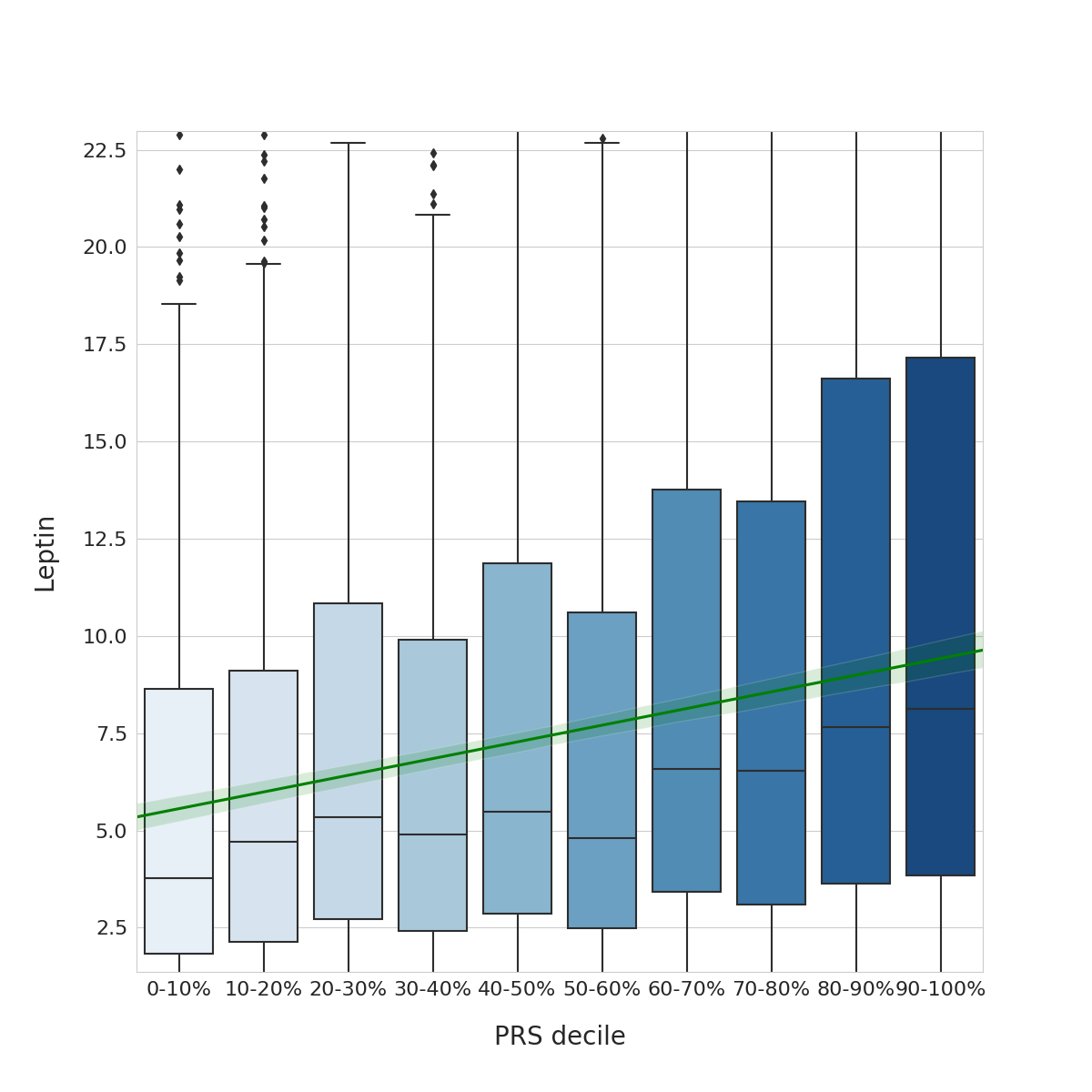

Supplement: S1 File — (ZIP) [file pone.0258748.s001.zip › Supplementary File 1/Leptin.png]

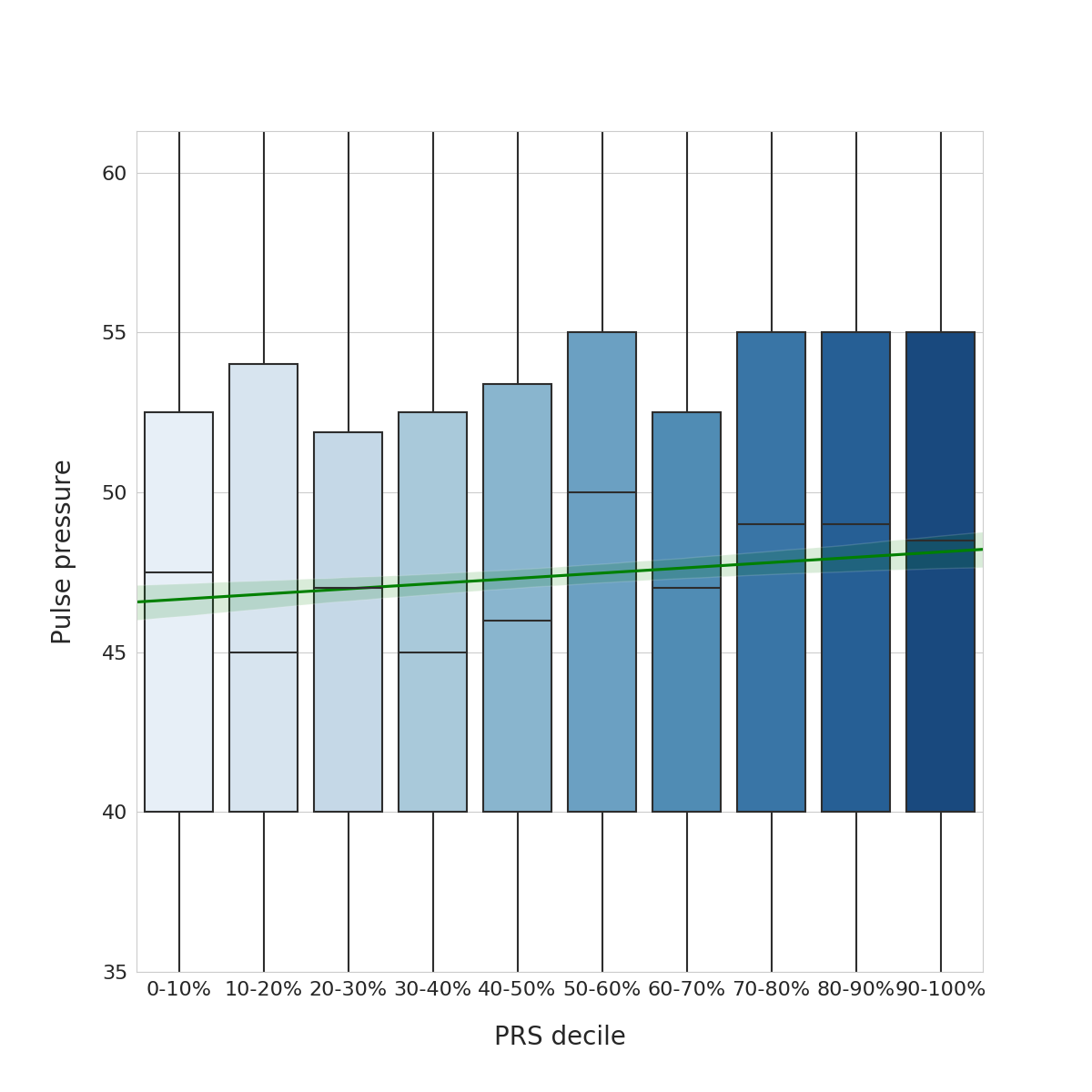

Supplement: S1 File — (ZIP) [file pone.0258748.s001.zip › Supplementary File 1/Pulse pressure.png]

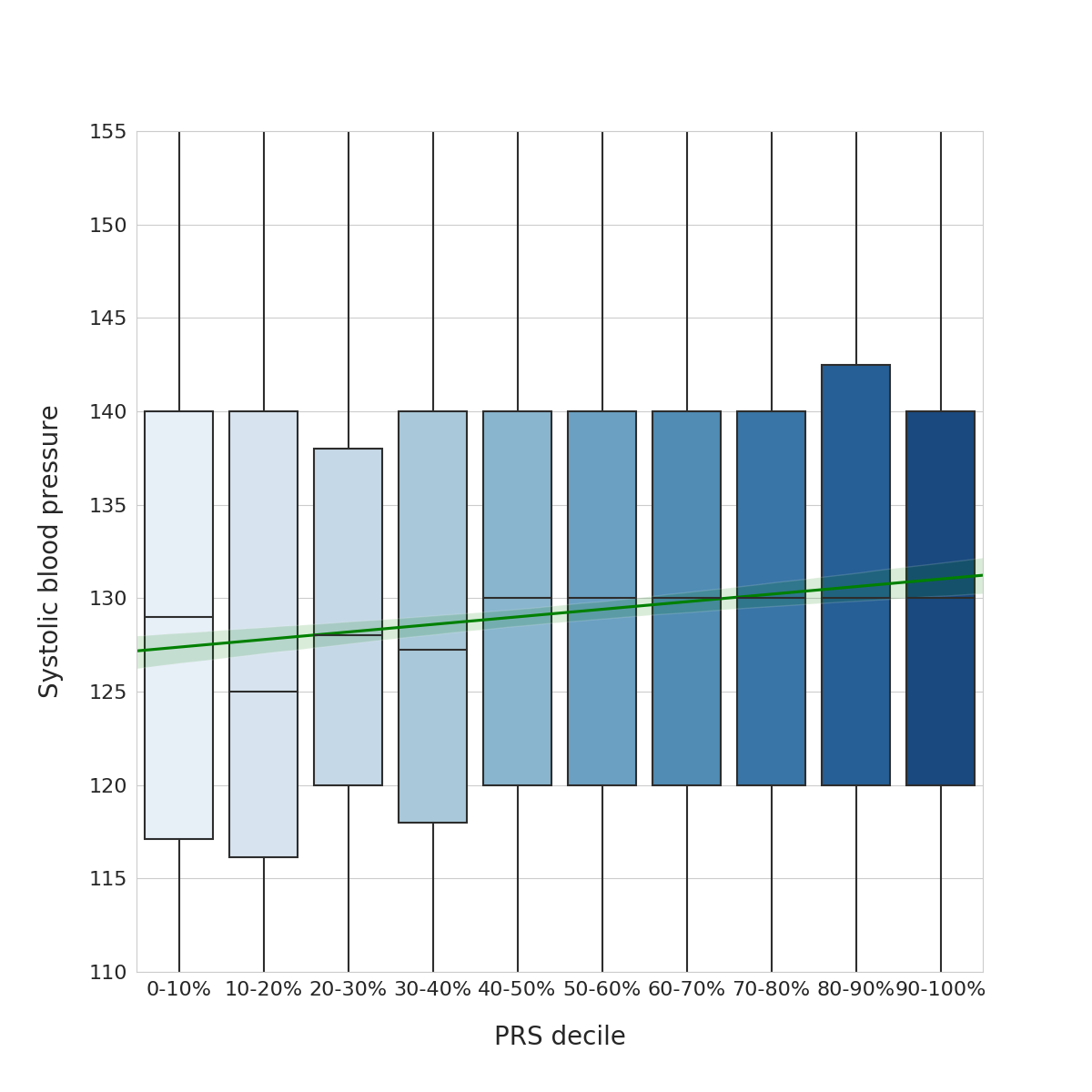

Supplement: S1 File — (ZIP) [file pone.0258748.s001.zip › Supplementary File 1/Systolic blood pressure.png]

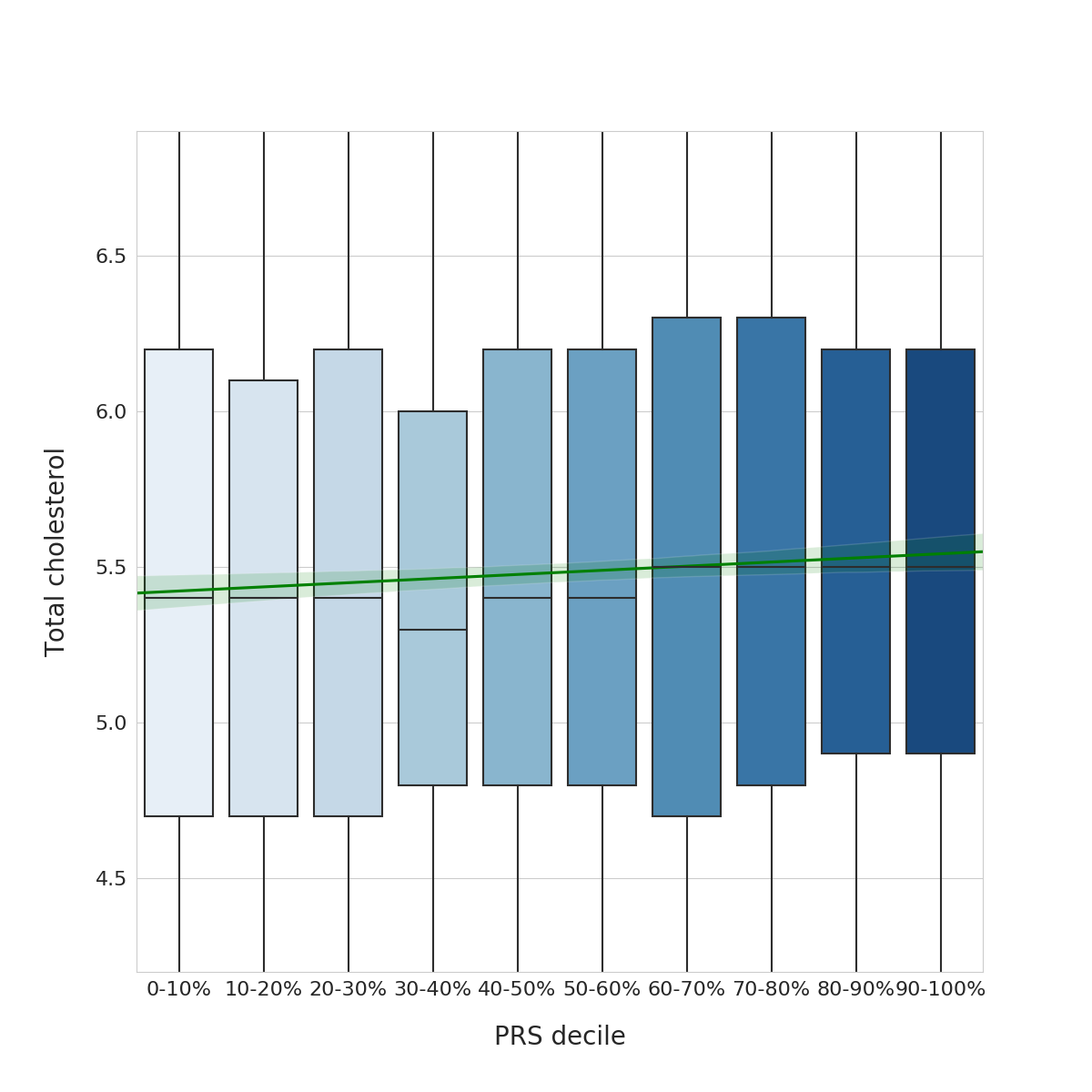

Supplement: S1 File — (ZIP) [file pone.0258748.s001.zip › Supplementary File 1/Total cholesterol.png]

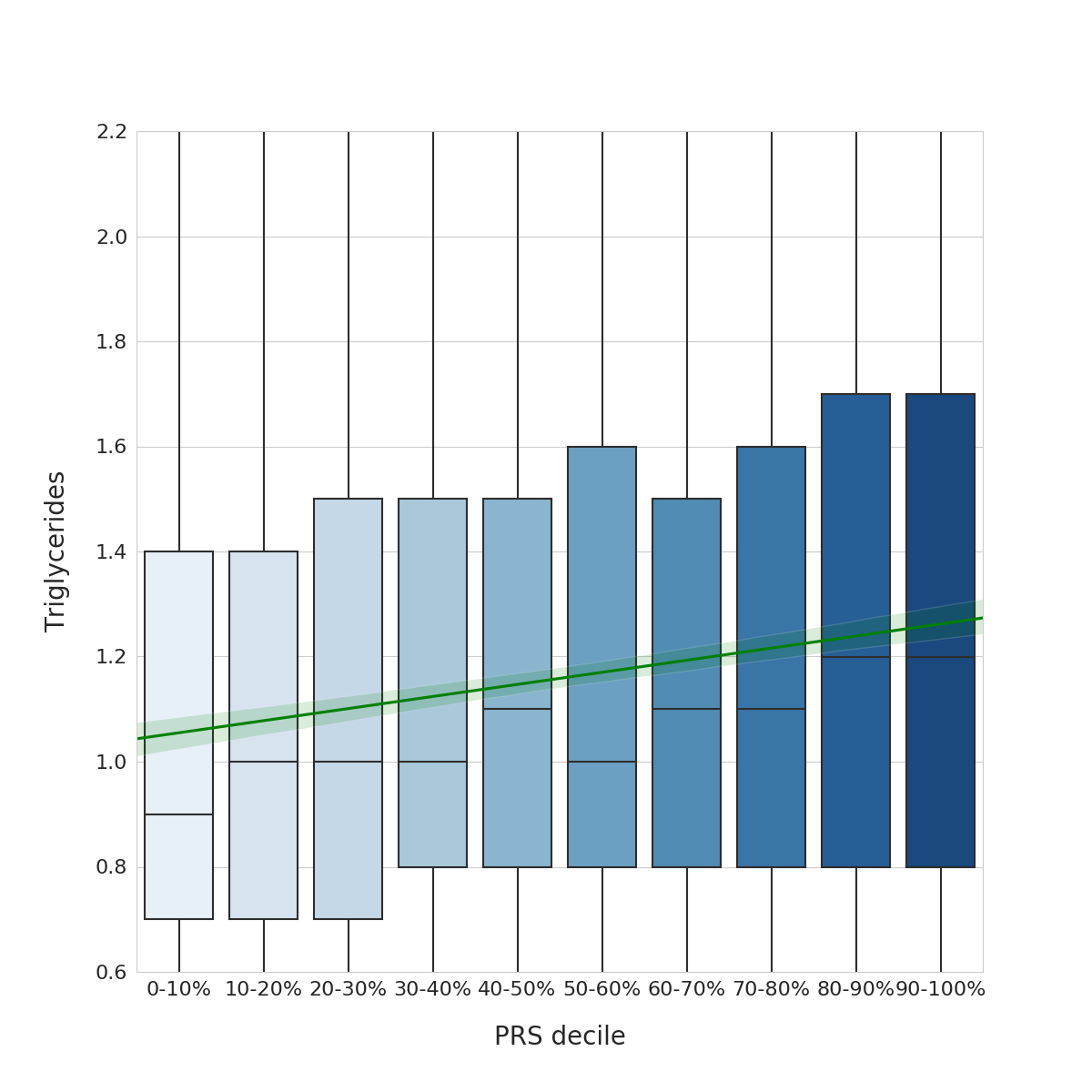

Supplement: S1 File — (ZIP) [file pone.0258748.s001.zip › Supplementary File 1/Triglycerides.png]

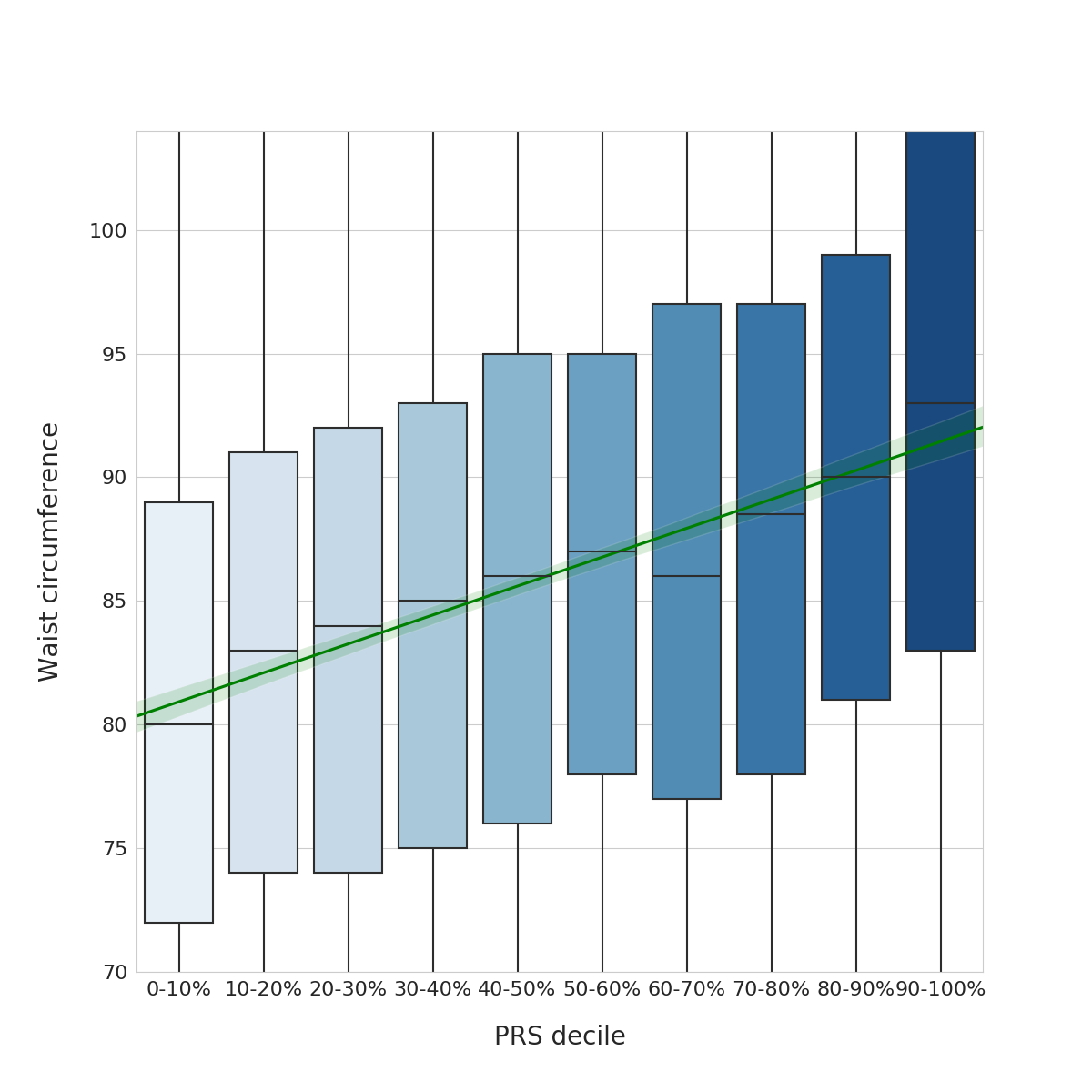

Supplement: S1 File — (ZIP) [file pone.0258748.s001.zip › Supplementary File 1/Waist circumference.png]

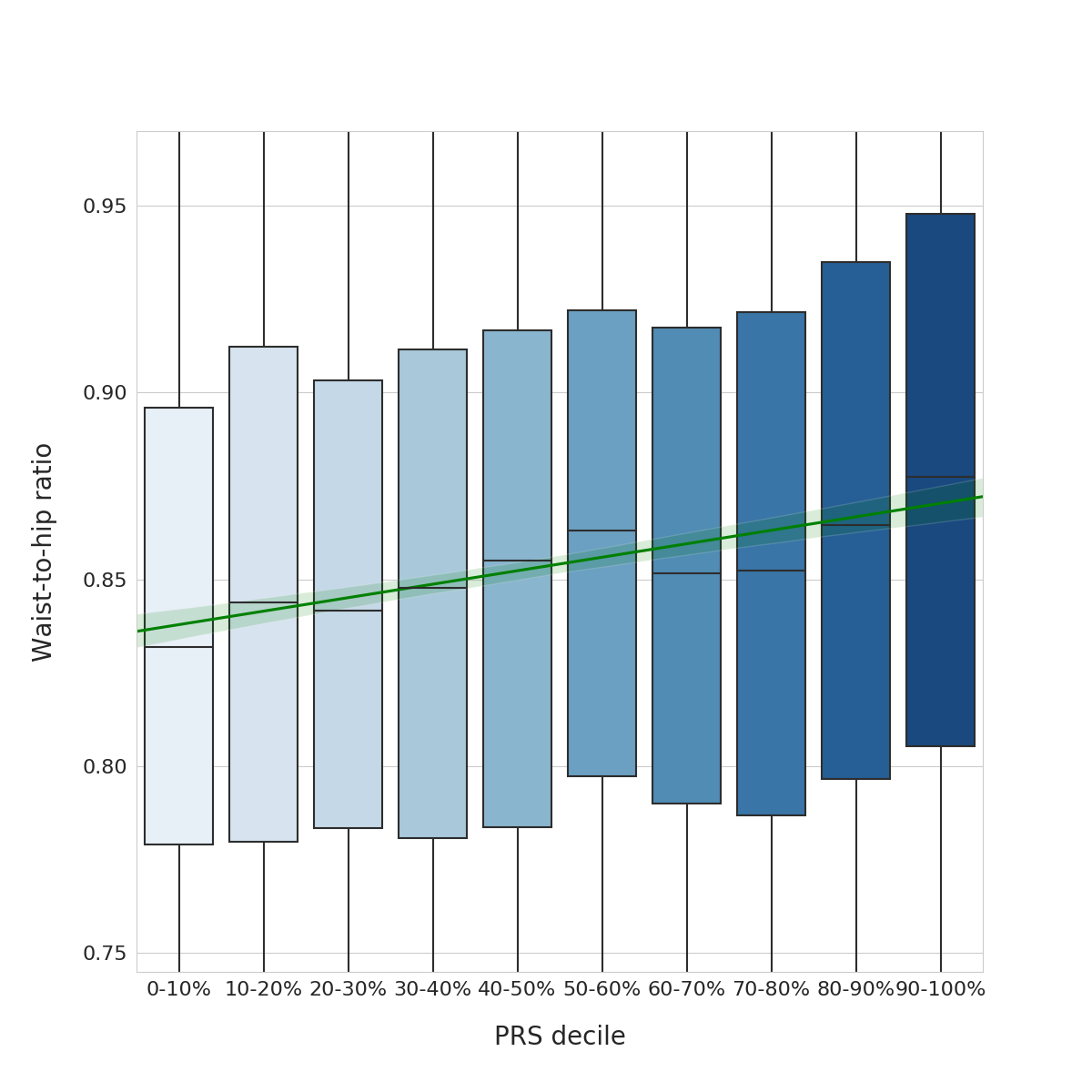

Supplement: S1 File — (ZIP) [file pone.0258748.s001.zip › Supplementary File 1/Waist-to-hip ratio.png]

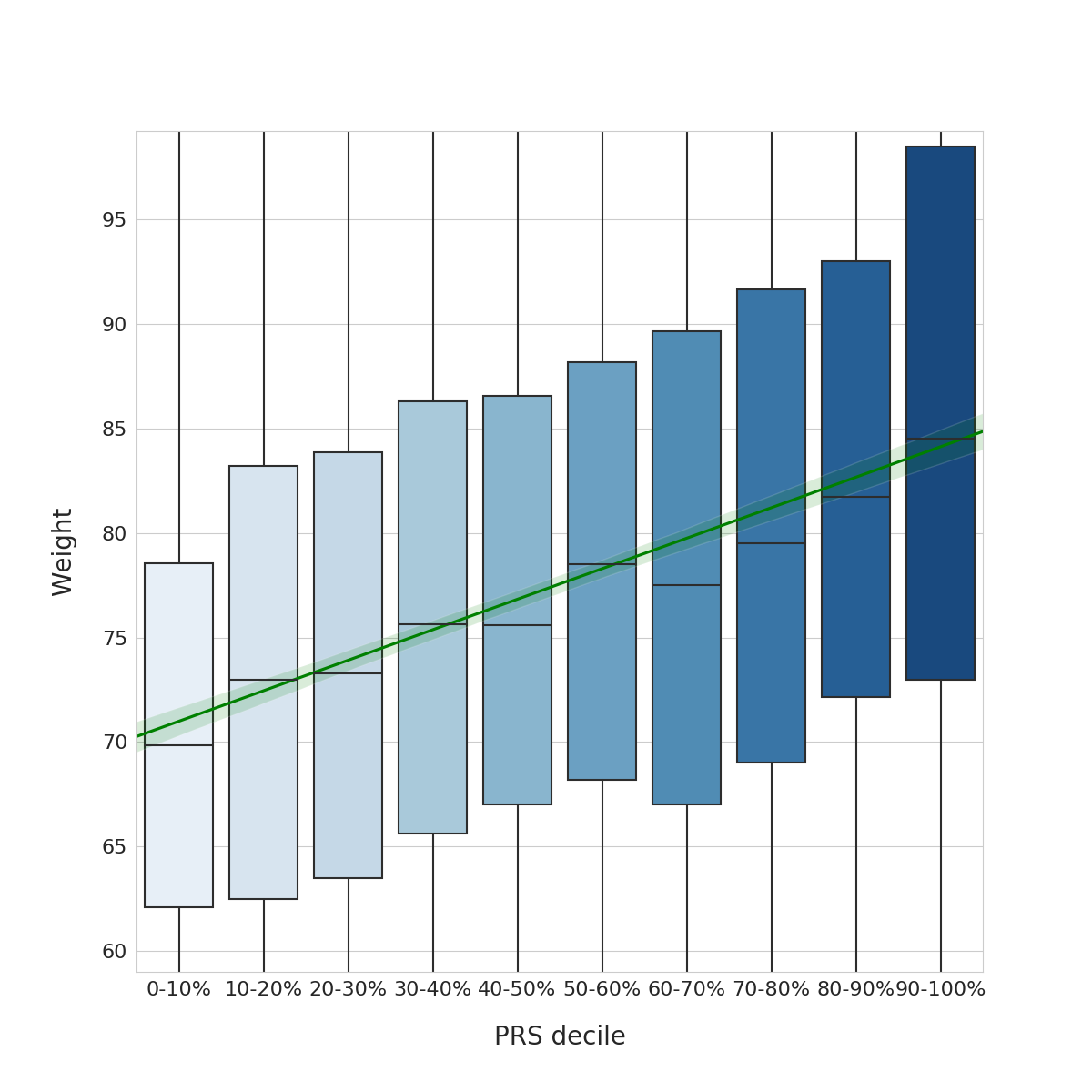

Supplement: S1 File — (ZIP) [file pone.0258748.s001.zip › Supplementary File 1/Weight.png]
