# Supplementary material for: Non-linear interaction between physical activity and polygenic risk score of body mass index in Danish and Russian populations
Source: PLoS One. 2021 Oct 18;16(10):e0258748. doi: 10.1371/journal.pone.0258748 (PMC8523041; doi:10.1371/journal.pone.0258748)
Supplement: S1 Fig — (DOCX) [file pone.0258748.s002.docx]

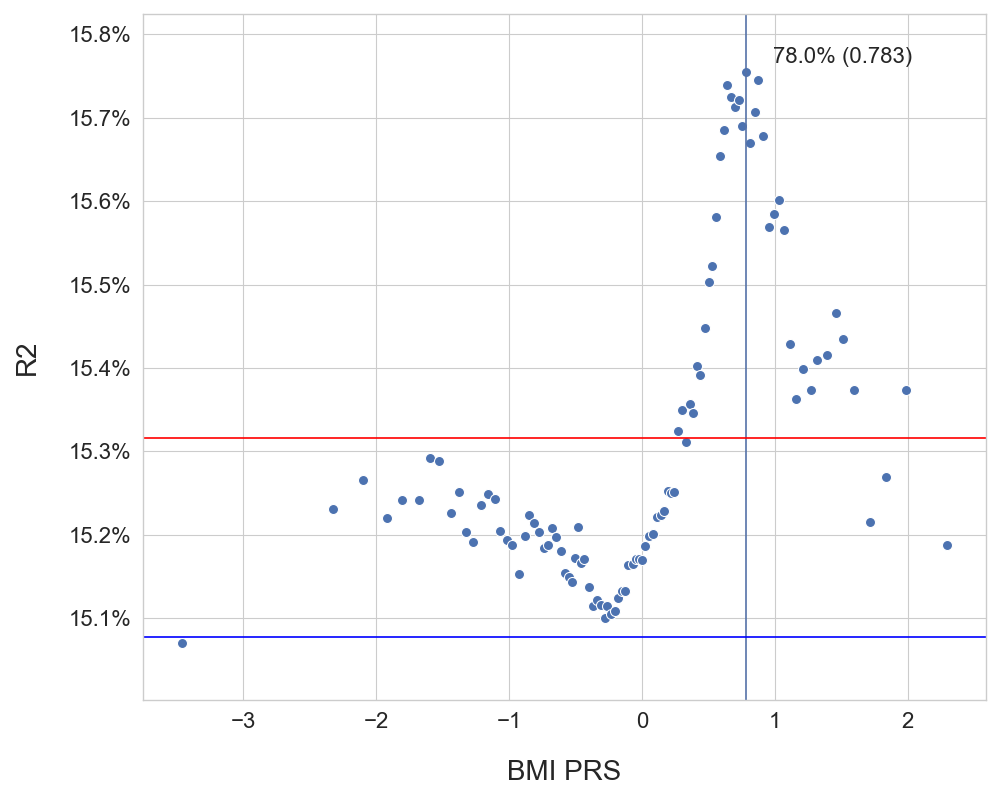


**S1 Fig. Performance of models using two groups of genetic risk for interaction with physical activity.**Each dot represents the performance of a different regression model with the outcome of BMI residuals and independent variables BMI PRS, physical activity, and an interaction between the physical activity and a given BMI PRS cut-off (linear regression *BMI_residuals ~ 0 + PRS + C(exercise) + C(exercise):I(PRS > i^th^ percentile)*). BMI residuals are calculated after regressing out age, sex, and intercept. Cut-offs are defined as i^th^ percentiles of the observed PRS, i=0,1,..,99. Higher R2 values correspond to a better model.
The best model was achieved when the individuals are combined in two groups with a cut-off at the 78^th^ percentile of BMI PRS, shown by a vertical line. For comparison, horizontal blue line shows the performance (15.08%) of the model without any interaction (linear regression *BMI_residuals ~ 0 + PRS + C(exercise)*), horizontal red line shows the performance (15.32%) of the model with linear interaction (linear regression *BMI_residuals ~ 0 + PRS + C(exercise) + C(exercise):PRS*).
