# Supplementary material for: Non-linear interaction between physical activity and polygenic risk score of body mass index in Danish and Russian populations
Source: PLoS One. 2021 Oct 18;16(10):e0258748. doi: 10.1371/journal.pone.0258748 (PMC8523041; doi:10.1371/journal.pone.0258748)
Supplement: S1 Appendix — (DOCX) [file pone.0258748.s005.docx]

**S1 Appendix. List of online supporting materials.**

1. The BMI PRS weight matrix and the interactive versions of Figs 2-4 used in this manuscript are available at <https://bmiprsxpa-pa7qfqmwhq-ew.a.run.app/>.
2. The code used for the analyses is available at GitHub at <https://github.com/borisevichdi/bmiprs-code>.
