# Supplementary material for: Non-linear interaction between physical activity and polygenic risk score of body mass index in Danish and Russian populations
Source: PLoS One. 2021 Oct 18;16(10):e0258748. doi: 10.1371/journal.pone.0258748 (PMC8523041; doi:10.1371/journal.pone.0258748)
Supplement: Attachment — Submitted filename: Plos One Review-June 7 (2021).docx [file pone.0258748.s006.docx]

**PLOS ONE:** Non-linear interaction between physical activity and polygenic risk score of body mass index in Danish and Russian populations

**Manuscript number:** PONE-D-21-17351

The present study analyzes the interaction between physical activity and polygenic risk scores for obesity. The authors identify a significant interaction between physical activity and a BMI polygenic risk score (PRS). The effect size of physical activity on BMI among the high-risk PRS was two times greater than the effect size in the low-risk PRS group. In my opinion, this is a methodologically strong paper that contributes to the evidence base in this area.

**Minor Comments**

Line 45: this section would benefit from describing other sources of missing heritability such as gene-gene interactions.

Line 186: is it possible to report the number and descriptive statistics of the individuals that were excluded based on missing data?

Line 256: I appreciate the authors’ creativity to remove *FTO* from the PRS and demonstrate the interaction with physical activity after excluding this variant. Has the interaction with physical activity been tested with any individual SNPs beyond *FTO*? I appreciate that this may be beyond the scope of this paper but may be worth investigating in future studies if it is a question of interest.

Line 259: I believe “There” should be revised to “These”

Line: 304: it would be informative for the reader to describe the nature of the interaction in the first paragraph of the discussion (e.g., physical activity was associated with a greater decrease in BMI among people with a higher PRS).

Line 315-316: the flow of this sentence could be improved, perhaps by adding “identify a” after “to”

**Major comments**

1. What is the clinical significance of the 78% cut-off? The authors state that this has important clinical implications but this is only shown empirically through the difference in effect size becoming two-fold greater in this group for the interaction. The clinical implications of this are not clear. Are there other cardiometabolic traits that increase significantly (e.g., past a certain threshold or markedly increase disease risk) in the high-risk PRS group? There may be more empirical support needed to justify that this threshold has important clinical implications.

2. BMI is an imperfect measure of obesity since it does not distinguish fat vs. fat free mass and this warrants mention in the limitations section.
